# Phytochemical profiling and fractionation of *Helianthemum lippii* extract versus silver nanoparticle-modified extract: assessment of photoprotective, anti-hemolytic, antibacterial, and anti-inflammatory properties

**DOI:** 10.3389/fchem.2024.1508707

**Published:** 2024-12-10

**Authors:** Ibtissam Laib, Djahra Ali Boutlilis, Huda Alsaeedi, David Cornu, Mikhael Bechelany, Ahmed Barhoum

**Affiliations:** ^1^ Department of Cellular and Molecular Biology, El Oued University, El Oued, Algeria; ^2^ Laboratory of Biodiversity and Biotechnology Applications in Agriculture, University of El Oued, El Oued, Algeria; ^3^ Laboratory of Biology, Environment and Health, Faculty of Natural and Life Sciences, University of El Oued, El Oued, Algeria; ^4^ Department of Chemistry, College of Science, King Saud University, Riyadh, Saudi Arabia; ^5^ Institut Européen des Membranes, IEM, University Montpellier, ENSCM, CNRS, Montpellier, France; ^6^ Functional Materials Group, Gulf University for Science and Technology (GUST), Mubarak Al-Abdullah, Kuwait; ^7^ Chemistry Department, NanoStruc Research Group, Faculty of Science, Helwan University, Cairo, Egypt

**Keywords:** Helianthemum lippii, green synthesis, bioactive fractions, anti-hemolytic activity, anti-inflammatory properties, anti-bacterial activity, biomedical applications

## Abstract

**Introduction:**

This study investigates the synthesis of silver nanoparticles (Ag NPs) using Helianthemum lippii extract and evaluates their photoprotective, anti-hemolytic, antibacterial, and anti-inflammatory properties compared to various extract fractions, including total aqueous extract (AE), flavonoid monoglycosides (FMG), flavonoid diglycosides/triglycosides (FDG/FTG), tannins (TN), and anthocyanins (AC). Helianthemum lippii is rich in bioactive compounds such as caffeic acid, p-coumaric acid, and gallic acid, known for their therapeutic potential. This study aims to determine whether embedding these phytochemicals into Ag NPs enhances their biomedical applications compared to the natural extract fractions.

**Methods:**

Ag NPs were synthesized using *Helianthemum lippii* extract through a green synthesis approach, and their physicochemical properties, including size and morphology, were characterized. High-performance liquid chromatography (HPLC) was used to identify key phytochemicals in the various extract fractions. Biological assays were conducted to assess photoprotective efficacy (sun protection factor, SPF), antibacterial activity (minimum inhibitory concentration, MIC), anti-inflammatory potential (percentage inhibition), and hemolytic properties, with sodium dodecyl sulfate (SDS) serving as a control.

**Results:**

HPLC analysis confirmed the presence of bioactive compounds, including caffeic acid, p-coumaric acid, and gallic acid, in the AE extract. The Ag NPs were spherical with an average diameter of 35 nm and had phytochemicals adsorbed on their surface. The AC fraction demonstrated the highest SPF (28.27), followed by TN (27.99), AE (23.20), and Ag NPs (22.50), while FMG exhibited a moderate SPF (19.39) compared to the commercial sunscreen Avene^®^ (40.00). Ag NPs exhibited superior antibacterial activity with MIC values of 0.2 mg/mL against *Pseudomonas aeruginosa* and 0.4 mg/mL against *Bacillus subtilis*, outperforming AE, which had a MIC of 2.81 mg/mL. Anti-inflammatory assays showed that Ag NPs achieved 79.8% inhibition at 400 μg/mL, surpassing AE (71.75%) and TN (67.9%), and were comparable to diclofenac (72.63%). Hemolysis assays revealed that Ag NPs induced only 1.35% hemolysis, lower than AE (1.91%) and significantly below SDS (90.48%).

**Discussion:**

The findings demonstrate that Helianthemum lippii-derived Ag NPs exhibit enhanced antibacterial, anti-inflammatory, and anti-hemolytic properties compared to the extract fractions. While the SPF of Ag NPs was slightly lower than the AC and TN fractions, their superior multifunctional bioactivities underscore their potential for various biomedical applications. The integration of phytochemicals into Ag NPs significantly enhances their therapeutic efficacy, making them promising candidates for advanced pharmaceutical formulations and topical protective agents.

## 1 Introduction

Phytochemicals are natural compounds synthesized by plants to defend against environmental threats such as pests and diseases. These bioactive molecules, including flavonoids, phenolics, and terpenoids, exhibit substantial medicinal potential and play a crucial role in disease prevention and management (Jameel and Yaser, 2020; Maghimaa and Alharbi, 2020; Nasrollahzadeh et al., 2019; Phillipson, 2001). For example, quercetin, a flavonoid found in citrus fruits, demonstrates potent antioxidant properties that reduce inflammation and lower the risk of chronic illnesses (Alsabri et al., 2013; Laib and Djahra, 2022), while phenolic compounds like epigallocatechin gallate (EGCG) from green tea are linked to anticancer activities and improved cardiovascular health. Similarly, terpenoids such as curcumin, derived from turmeric, are renowned for their anti-inflammatory effects and potential in preventing Alzheimer’s disease (Wei et al., 2015; Zhang et al., 2016). Investigating these compounds, particularly in plants like *Helianthemum lippii*, is vital for exploring their therapeutic properties. Advanced fractionation techniques are used to isolate and purify these phytochemicals, separating bioactive constituents from less beneficial substances and revealing synergistic interactions that enhance their overall effects (Djemam et al., 2020; Plescia et al., 2022). Moreover, combining fractionated plant extracts with nanotechnology to develop bioactive nanoparticles opens novel avenues for creating innovative treatments that harness the full potential of these natural compounds (Laib et al., 2024a; Tunç, 2024).

Plant extracts, rich in phytochemicals, serve as eco-friendly and effective reducing and stabilizing agents in the synthesis of Ag NPs, enhancing their biological properties and broadening their therapeutic potential. The resulting Ag NPs exhibit significant antimicrobial, anti-inflammatory, and antioxidant effects, making them highly suitable for various medical applications (Grodzicki et al., 2024; Ibtissam and Boutlilis, 2023). For example, *Curcuma longa* (turmeric), which contains curcumin, known for its potent anti-inflammatory and antioxidant properties, enhances the antibacterial activity of Ag NPs, showing promise in wound healing and infection control (Khan et al., 2019; Maghimaa and Alharbi, 2020). Similarly, *Zingiber officinale* (ginger) provides anti-inflammatory benefits, adding a dual therapeutic effect when incorporated into Ag NP synthesis (Venkatadri et al., 2020). Additionally, *Azadirachta indica* (neem) extract facilitates the synthesis of Ag NPs with notable antifungal and antibacterial properties, broadening their application in infection management (Alharbi and Alsubhi, 2022; Laib et al., 2023), while *Allium sativum* (garlic) produces Ag NPs with remarkable antibacterial effects (Ohiduzzaman et al., 2023). By combining the bioactivity of plant-derived compounds with nanotechnology, this approach not only amplifies the functional properties of Ag NPs but also offers a sustainable platform for developing innovative therapeutic agents that address diverse global healthcare challenges.

*Helianthemum lippii* known for its rich content of flavonoids, polyphenols, and tannins. It has garnered attention in traditional medicine for its therapeutic potential (Alharbi and Alsubhi, 2022; Laib and Djahra, 2022). Its antioxidant, anti-inflammatory, and antimicrobial properties make it a promising candidate for various health applications. However, its photoprotective properties, particularly its ability to counteract UV-induced damage, remain largely unexplored (Alaguvel and Sundaramurthy, 2024; Alharbi and Alsubhi, 2022). This presents a significant research gap, offering opportunities to investigate how *H. lippii* can be combined with Ag NPs to enhance its therapeutic efficacy. The phytochemicals in *H. lippii* not only contribute to its antioxidant properties but may also play a role in stabilizing and improving the bioavailability of Ag NPs, potentially enhancing their therapeutic effects. Furthermore, the plant’s anti-hemolytic activity is particularly relevant, as oxidative stress can cause hemolysis, a critical health issue. While Ag NPs have been shown to prevent hemolysis under oxidative stress, the combined effects of *H. lippii* extracts and Ag NPs on red blood cell protection remain underexplored. Investigating these synergistic effects could provide new insights into their therapeutic potential and open up innovative strategies that combine natural and nanomaterial-based approaches to address oxidative stress and cellular damage.

This study investigates the photoprotective, anti-hemolytic, antibacterial, and anti-inflammatory properties of Ag NPs synthesized using *H. lippii* extract, compared to various extract fractions: total aqueous extract (AE), flavonoid monoglycosides (FMG), flavonoid diglycosides/triglycosides (FDG/FTG), tannins (TN), and anthocyanins (AC). The aim of the study is to evaluate how these Ag NPs, combined with plant extracts, enhance therapeutic effects, focusing on their ability to mitigate UV-induced damage, prevent hemolysis, and modulate inflammatory responses. The methodology includes standardized assays such as the agar diffusion method for antibacterial activity, using Gram-positive bacteria (*Bacillus subtilis* and *Staphylococcus aureus*) and Gram-negative bacteria (*Escherichia coli* and *Pseudomonas aeruginosa*) for comparison. Anti-hemolytic activity was assessed using red blood cell protection, with sodium dodecyl sulfate (SDS) as a positive control. Anti-inflammatory assays measured the modulation of pro-inflammatory cytokines, with diclofenac serving as the standard anti-inflammatory control. For photoprotection, the sun protection factor (SPF) was used to evaluate the protective effect against UV radiation, with Avene^®^ as the commercial standard. The research is of significant importance as it explores the synergistic effects of Ag NPs and *H. lippii* bioactive fractions, contributing to the growing field of natural product pharmacology. This work aims to optimize the use of Ag NPs and *H. lippii* bioactive fractions in medicine, particularly for therapeutic and cosmetic applications, by filling gaps in the current scientific understanding of their combined effects.

## 2 Experimental

### 2.1 Chemicals and reagents

All chemicals and reagents utilized in this study were of analytical grade and procured from Sigma-Aldrich (St. Louis, MO, United States). The primary chemical employed was silver nitrate (AgNO₃, 99.9%), which played a crucial role in the synthesis of Ag NPs. Additionally, various other chemicals were necessary for the experimental protocols, including sodium dodecyl sulfate (C₁₂H₂₅NaO₄S, 98%), potassium dihydrogen phosphate (KH₂PO₄, 99.5%), dibasic potassium phosphate (K₂HPO₄, 99.95%), ethylenediaminetetraacetic acid (EDTA, 99.0%), methanol (CH₃OH, ≥99.9%), hydrochloric acid (HCl, ≥37%), acetone (C₃H₆O, ≥99.5%), ethyl acetate (C₄H₈O₂, ≥99.5%), n-butanol (C₄H₁₀O, ≥99.5%), Tannins (CH₂Cl₂, ≥99.9%), dimethyl sulfoxide (DMSO, C₂H₆OS, 99.9%), ethyl ether (CH_3_-CH_2_-O-CH_2-_CH_3,_ ≥99.5%), Petroleum Ether (CH_3_-CH(CH_3_)-CH_2_-CH_3,_ 95%–99%) and diclofenac (C₆H₄Cl₂-NH-C₆H₄-COOH, ≥98%). These reagents were used for various assays and analyses throughout the study to ensure the accuracy and reliability of the results.

### 2.2 Bacterial strains

The bacterial strains utilized for evaluating antibacterial activity included two Gram-positive bacteria, namely, *B. subtilis* ATCC 6633 and *S. aureus* ATCC 6538, as well as two Gram-negative bacteria, *E. coli* ATCC 8737 and *Pseudomonas aeruginosa* ATCC 9027. These strains were obtained from the Pasteur Institute’s laboratory in Algiers, Algeria, and were maintained under appropriate conditions to ensure their viability and stability prior to experimentation.

### 2.3 Collection and preparation of H. lippii extract

The aerial parts of *H. lippii* were meticulously collected in March 2022 from the pristine Elhamadin region in El-Oued province, Algeria (33°35′00″N, 6°56′33″E). Additionally, a voucher specimen was deposited under the number PS/He. li 2014 at the Herbarium of the Biology Department, University of Hamma Lakhdar, El Oued, Algeria, ensuring proper documentation and availability for reference and verification. The plant species was identified and authenticated by an expert botanist. Following collection, the plant material was carefully stored in a cool, dry environment shielded from light to preserve its phytochemical integrity. After drying, 10 g of the plant material was soaked in 100 mL of distilled water and left at room temperature in the dark for 24 h to facilitate the extraction of bioactive compounds. The resulting mixture was then filtered through a Whatman No. 1 filter paper, and the filtrate was evaporated at 40°C using a rotary evaporator to remove excess water. The dry extract was weighed and subsequently stored at 4°C for further analysis, ensuring that it retained its bioactive properties for subsequent experimental applications (Bouabid et al., 2020).

### 2.4 HPLC analysis

The phenolic profile of the crude extract was meticulously analyzed using a state-of-the-art Shimadzu LC20 AL high-performance liquid chromatography (HPLC) system, which is integrated with an advanced UV-Vis detector (SPD 20A) to ensure precise detection of phenolic compounds. The separation process was executed on a high-resolution Shim-pack VP-ODS C18 column (4.6 mm × 250 mm, 5 µm particle size), ensuring optimal resolution and efficiency. A universal Hamilton 25 µL injector was employed to guarantee consistency in sample introduction. The mobile phase consisted of acetonitrile and 0.1% acetic acid, utilized in a gradient elution mode optimized specifically for reverse-phase chromatography. This approach effectively separates non-polar and moderately polar phenolic compounds, allowing for a thorough analysis of the extract. The flow rate was meticulously controlled at 1 mL/min, and an injection volume of 20 µL was used to enhance precision and sensitivity in the detection of phenolic compounds. Phenolic compounds were monitored at an optimal wavelength of 268 nm, which was selected based on the characteristic absorbance of the target analytes. Identification of the compounds was achieved by comparing their retention times against known standards, which were prepared under identical conditions. Quantification of the phenolic compounds was expressed as micrograms per gram of dry extract (µg/g), providing a comprehensive insight into the phenolic composition of the extract. In addition, calibration curves for each standard phenolic compound were constructed to facilitate accurate quantification.

### 2.5 Extraction and fractionation of flavonoids

Flavonoids were extracted from finely ground, dried *H. lippii* using an optimized protocol based on the methods of Markham (1982) and Bruneton (1993). The plant material was initially extracted 10 g with 100 mL of 85% methanol at a concentration of 10% w/v. After extraction, the solution was filtered under reduced pressure using a Büchner funnel, and the extract was concentrated at 35°C using a rotary evaporator (Rota Vapor, Büchi 461, Germany). The resulting aqueous phase was stored at 40°C for 48 h to facilitate molecular diffusion, after which it was filtered again to remove any remaining solid particles. To further fractionate the flavonoids, the aqueous phase underwent successive solvent extractions. The first extraction involved washing the extract three times with 150 mL of petroleum ether (v/v) to remove lipids, waxes, and chlorophyll, resulting in a purified aqueous fraction. The next step involved extracting flavonoid aglycones, including methoxylated aglycones, by treating the aqueous phase with ethyl ether. The remaining aqueous phase was then extracted three times with ethyl acetate to isolate monoglycosides and some aglycones. Finally, n-butanol was used to recover di- and triglycosides. The flavonoid fraction (FDG/FTG) were concentrated under reduced pressure at 35°C and lyophilized for 24 h using an ALPHA 1-2 LD lyophilizer (Fisher Bioblock). Lyophilization ensured the preservation of the compounds’ solubility and chemical integrity. The yield of each fraction was calculated and expressed as grams of lyophilized product per 100 g of dry plant material (Bruneton, 1993; Markham, 1982).

### 2.6 Extraction of anthocyanin

According to Vayupharp and Laksanalamai, 2015, 12 g of plant material were steeped in 50 mL of methanol acidified with 50 µL of 0.1% HCl (v/v). The pH of this solution is approximately 2–3, providing the acidic environment necessary for efficient anthocyanin extraction, stirred in the dark at room temperature for 20 h for the extraction of anthocyanins. The resulting mixture was filtered over Whatman No. 1 filter paper and the filtration residue was rinsed with 50 mL of methanol containing 0.1v/v% HCl to ensure maximum recovery of anthocyanins. The combined filtrates were evaporated to dryness in a rotary evaporator at 30°C. The resultant residue was dissolved in 50 mL of deionized water containing 0.01 v/v% HCl, and centrifugation was conducted at 3,000 rpm for 15 min. The supernatant obtained now contained anthocyanins and was ready for further analysis (Vayupharp and Laksanalamai, 2015).

### 2.7 Extraction and fractionation of tannins

Tannins extraction was performed based on a modified procedure of Sepperer and Tondi (2018). About 30 g of dried *H*. *lippii* was allowed to soak in 300 mL of water-acetone mixture (7:3 v/v%) at room temperature for 3 days with periodic stirring to increase the power of extraction. Acetone was removed after maceration by filtering the solution through Whatman No. 1 filter paper and retaining the aqueous phase. This phase was then extracted twice with 180 mL portions of ethyl acetate to effectively separate the tannins. The organic phases from both extractions were combined and concentrated at 40°C in a rotary evaporator to a viscous residue. The tannin-rich extract was dried under reduced pressure and stored in airtight containers at 4°C for further biological evaluation (Sepperer and Tondi, 2018).

### 2.8 Synthesis and characterization of Ag NPs modified using *H. lippii* extract

A 1.0 mM AgNO_3_ solution was prepared as the silver ion source. To this solution, 10 mL of *H. lippii* extract was added to 90 mL of the AgNO₃ solution under continuous stirring. The reaction was conducted in the dark at 60°C for 24 h, during which a color change from pale yellow to brown was observed, indicating the formation of Ag NPs. After the reaction, the mixture was allowed to cool, and the resulting nanoparticles were separated by centrifugation at 10.000 rpm for 20 min. The nanoparticles were then washed with distilled water and ethanol to remove any unreacted materials and by products, followed by drying at 60°C to obtain a fine powder (Laib et al., 2023).

Characterization of the synthesized Ag NPs involved several techniques. UV-Vis spectroscopy was performed using a Shimadzu UV-1800 spectrophotometer, with measurements taken in the range of 200–800 nm at room temperature. The absorbance peak in this range confirmed nanoparticle formation and was used to estimate their size based on the surface plasmon resonance (SPR) effect. X-ray diffraction (XRD) analysis was conducted using a PROTO AXRD system (30 kV, 20 mA, wavelength of 0.154,281 Å) to determine the crystallinity and phase composition of the nanoparticles. XRD patterns were recorded at a scanning rate of 0.05° per minute over a 2θ range of 20°–80°. For scanning electron microscopy (SEM) analysis, a thin layer of the dried Ag NPs was deposited on an SEM holder. The SEM images were captured to provide detailed insights into the morphology of the nanoparticles. Energy-dispersive X-ray (EDX) spectroscopy was conducted in conjunction with SEM to provide qualitative and quantitative data on the elemental composition of the synthesized Ag NPs, ensuring the presence of silver and confirming the successful modification with *H. lippii* extract.

### 2.9 Anti-inflammatory activity test

The anti-inflammatory activity of Ag NPs, the total aqueous extract (AE), and fractions from *H*. *lippii* (FMG, FDG/FTG, TN, and AC) was evaluated using the egg albumin denaturation inhibition method. In this assay, a total volume of 2.8 mL of phosphate buffer (pH 6.4) was mixed with 200 µL of fresh hen’s egg albumin and 2 mL of sample solutions at varying concentrations (100, 200, and 400 μg/mL) or a standard diclofenac solution at the same concentration. Distilled water was used as the negative control. The reaction mixtures were incubated at 37°C for 15 min to allow for proper interaction, followed by heating at 70°C for 5 min to induce albumin denaturation. After cooling to room temperature, the absorbance of the resulting solutions was measured at 660 nm using a UV-visible spectrophotometer, with phosphate buffer serving as the blank. The percentage of inhibition of egg albumin denaturation was calculated using the formula:

$$Inhibition\;of\;Albumin\;denaturation\;\left(\%\right)=\frac{Acontrol-A\;sample\;}{A\;control}$$

where A control denotes the absorbance of the control (distilled water), and *A sample* represents the absorbance with *H. lippii*, Ag NPs or fractions compounds. This calculation provided a quantitative measure of the anti-inflammatory activity of the extracts and Ag NPs, allowing for comparison with the standard drug, diclofenac (Naveed et al., 2022).

### 2.10 Hemolytic activity

The hemolytic activity of Ag NPs, the total aqueous extract (AE), and fractions from *H. lippii* (FMG, FDG/FTG, TN, and AC) was assessed following the method described by Vinjamuri et al. (2015). Fresh blood (5 mL) was collected and treated with 5.4 mg of EDTA to prevent coagulation. The blood was then centrifuged at 1,000 rpm for 10 min at 4°C. The erythrocytes were washed three times with phosphate-buffered saline (PBS) and incubated at 4°C for 6 h to stabilize the cells. Test samples were prepared at concentrations of 25, 50, and 100 μg/mL 100 μL of each test sample was mixed with 50 µL of erythrocyte suspension in a 10-fold dilution series in a total volume of 1 mL. Positive and negative controls consisted of 100 µL of 1X PBS (representing normal conditions) and 100 µL of 1% sodium dodecyl sulfate (SDS) (inducing complete lysis), respectively. The reaction mixtures were incubated in a water bath at 37°C for 60 min. After incubation, 850 µL of 1X PBS was added to adjust the final volume to 1 mL. The samples were centrifuged at 3,000 rpm for 3 min, and the supernatant was collected. Hemoglobin concentration in the supernatant was measured using a spectrophotometer at 540 nm. The degree of hemolysis was calculated using the formula:

$$\text{Hemolysis }\left(\%\right)=\frac{Acontrol-A\;sample\;}{A\;control}$$

where A control denotes the absorbance of the control (PBS) and *A sample* represents the absorbance with *H. lippii*, Ag NPs or fractions compounds. To ensure accuracy, each test was performed in triplicate.

### 2.11 Sun protection factor (SPF) calculation

The photoprotective efficacy of the Ag NPs, the total aqueous extract (AE) and fractions from *H. lippii* (FMG, FDG/FTG, TN, and AC) was evaluated by measuring their SPF using spectrophotometric analysis. Each sample was prepared at 1 mg/mL in ethanol for consistency. Absorbance was measured between 290 and 320 nm, with 5 nm intervals. Avene^®^, a commercially available sunscreen, was used as a positive control. The SPF values were calculated using the formula outlined by Rudrappa et al. (2023):

$$\text{SPF }\text{Spectrophotometric}=CF\times \sum_{290}^{320}\times EE\;\left(\lambda \right)\times I\;\left(\lambda \right)\times DO\;\left(\lambda \right)$$

where: CF; correction factor (10). EE; erythemogenic effect of radiation with wavelength (λ) nm. I; solar intensity spectrum (λ) nm. DO (λ); spectrophotometric absorbance values at wavelength. The values of EE (λ) × I (λ) are constants. To ensure accuracy, each test was performed in triplicate.

### 2.12 Antibacterial activity

The antibacterial activity of Ag NPs, the total aqueous extract (AE), and fractions from *H*. *lippii* (FMG, FDG/FTG, TN, and AC), was evaluated using the agar diffusion method. Bacterial strains were cultured on nutrient agar for 24 h at 37°C to reach the stationary phase of growth. Suspensions of bacterial cells, standardized to 10⁶ colony-forming units per mL, were evenly spread onto Mueller Hinton agar plates using sterile swabs. Discs (6 mm diameter) were impregnated with 10 µL of each sample dissolved in 5 v/v% DMSO at varying concentrations: 20 mg/mL for the plant extract, 10 mg/mL for bioactive compounds, and 3 mg/mL for Ag NPs (Belhaoues et al., 2020). Amoxicillin (10 μg/mL) and cephalexin (30 μg/mL) were used as positive controls, while DMSO served as the negative control. The plates were incubated at 37°C for 24 h, and inhibition zones were measured to assess antibacterial effectiveness. To ensure accuracy, each test was performed in triplicate.

For determining the Minimum Inhibitory Concentration (MIC), a broth macro-dilution method was employed. Stock solutions of plant extracts, bioactive compounds, and Ag NPs were prepared and diluted in Mueller Hinton Broth (MHB). Sterile test tubes containing 0.2 mL of bacterial inoculum were inoculated with 2 mL of each sample at various concentrations, yielding a final volume of 4 mL per tube. Controls included MHB with bacterial inoculum and MHB without inoculum. The tubes were incubated at 37°C for 24 h to identify the lowest concentration that inhibited visible bacterial growth (Verma et al., 2016).

### 2.13 Statistical analysis

Statistical analysis was performed using OriginPro (2018 64-bit) and SPSS Statistics Version 26. Data obtained from the anti-inflammatory, hemolytic activity, and SPF experiments were expressed as means ± standard deviation (SD) for n = 3 replicates. One-way ANOVA was used to evaluate significant differences between treatments, and Tukey’s B test was applied for *post hoc* analysis to determine specific group differences. A significance level of *p* ≤ 0.05 was set for all analyses to ensure statistical relevance.

## 3 Results and Discussion

### 3.1 Yields and distribution of bioactive compounds in *H. lippii*

The extraction of bioactive compounds from *H. lippii* yielded an aqueous extract (AE) with a percentage yield of 9.83%, indicating a substantial content of bioactive constituents (Table 1). The AC fraction was the most abundant, comprising 5.66% of the extract, highlighting its strong antioxidant potential and role in photoprotection and oxidative stress reduction. The FMG fraction (0.75%) and FDG/FTG fraction (0.99%), along with TN fraction (0.71%), had lower yields but still contributed to the extract’s bioactivity. These minor fractions, though present in smaller quantities, likely play complementary roles, enhancing the extract’s overall therapeutic effects. Despite these smaller quantities, these compounds still contribute to the plant’s bioactivity, likely playing a complementary role in its therapeutic effects (Fellah et al., 2019).

**TABLE 1:** Yields and distribution of bioactive compounds in *H. Lippii extract*.

Bioactive compound	Yields (%)
Aqueous extract (AE)	9.83 ± 0.24
Flavonoid Monoglycosides (FMG)	0.75 ± 0.06
Flavonoid diglycosides/triglycosides (FDG/FTG)	0.99 ± 0.09
Tannin (TN)	0.71 ± 0.02
Anthocyanins (AC)	5.66 ± 0.43

### 3.2 HPLC quantitative analysis

**FIGURE 1:**
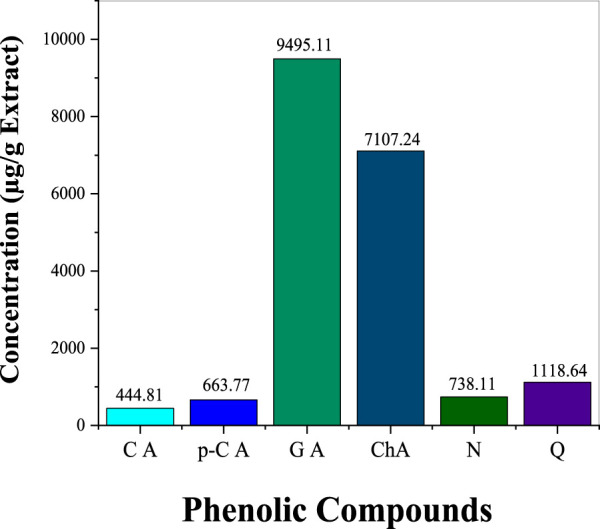
Concentration of key phenolic compounds in *H. lippii* aqueous extract: CA (Caffeic Acid), p-C (p-Coumaric Acid), GA (Gallic Acid), ChA (Chlorogenic Acid), N (Naringin), Q (Quercetin)

**FIGURE 2:**
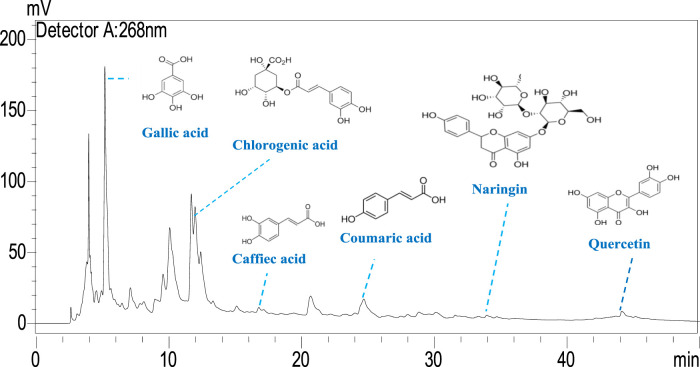
HPLC chromatogram of the *H. lippii* aqueous extract.

The phenolic profile of *H. lippii* extract demonstrates a rich concentration of bioactive compounds (Figure 1), with gallic acid (9,495.11 μg/g) and chlorogenic acid (7,107.24 μg/g) as the predominant constituents. These compounds, renowned for their potent antioxidant and anti-inflammatory properties, play a pivotal role in mitigating oxidative stress and inflammation, establishing them as primary contributors to the extract’s bioactivity (Qi et al., 2023). Supporting these major phenolics, the extract also contains quercetin (1,118.64 μg/g), p-coumaric acid (663.77 μg/g), and naringin (738.19 μg/g) (Figure 2). While present in lower concentrations, these secondary phenolics enhance the therapeutic potential of the extract. Quercetin’s antioxidant effects and the anti-inflammatory properties of p-coumaric acid and naringin further amplify the extract’s efficacy (Al-Khayri et al., 2022; Kleemann et al., 2011). Despite the absence of vanillic acid, rutin, and vanillin, the robust presence of other phenolics ensures the extract’s therapeutic promise remains intact. The synergy among gallic acid, chlorogenic acid, and quercetin likely underpins the extract’s ability to counter oxidative damage, prevent hemolysis, and reduce inflammation (Laib et al., 2024b; Su et al., 2021). This highlights the therapeutic potential of *H. lippii* extract, particularly in mitigating oxidative stress, enhancing skin protection, and supporting hematological health (Bedouhene et al., 2024; Laib et al., 2024a).

### 3.3 Characteristics of Ag NPs

**FIGURE 3:**
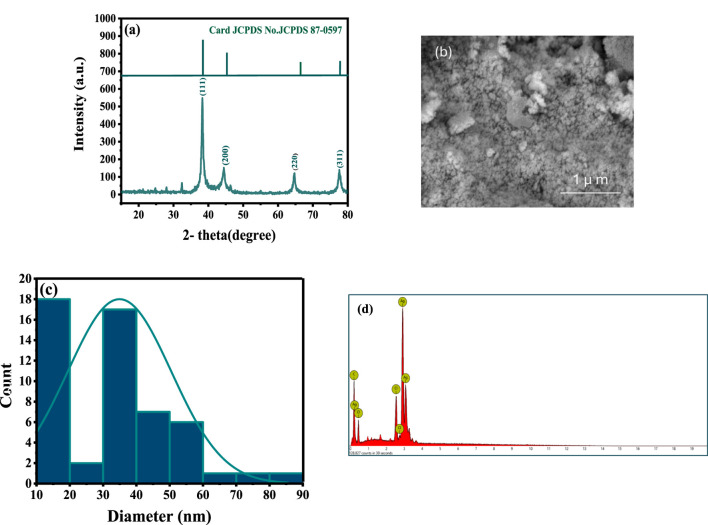
Characteristics of the synthesized Ag NPs coated with the phytochemical of: **(A)** XRD showing the crystalline structure, **(B)** SEM image, **(C)** Size distribution analysis, and **(D)** SEM-EDX.

Ag NPs were characterized to evaluate their structural, compositional, and morphological properties. XRD analysis confirmed their crystalline nature, showing peaks at 38.2°, 44.4°, 64.7°, and 77.5° (Figure 3A), corresponding to the (111) (200) (220), and (311) planes of a face-centered cubic (fcc) structure, consistent with metallic silver (JCPDS Card No. 04–0,783). The average crystallite size, calculated using the Scherrer equation, was 12.84 nm (Barhoum et al., 2016; Laib et al., 2024b). SEM imaging revealed a spherical morphology with an average particle size of 35 nm, indicating uniformity and stability (Barhoum et al., 2015; Figure 3B). Size distribution analysis supported these findings, showcasing a consistent particle size (Figure 3C). SEM-EDX analysis confirmed the elemental composition, with silver (71.83 wt%) being predominant, along with oxygen (12.50 wt%) and carbon (15.67 wt%) (Figure 3D). These additional elements were attributed to phytochemical residues, likely flavonoids and polyphenols from the plant extract, which acted as stabilizing and capping agents to prevent aggregation and enhance biological activity.

### 3.4 Anti-inflammatory activity

**FIGURE 4:**
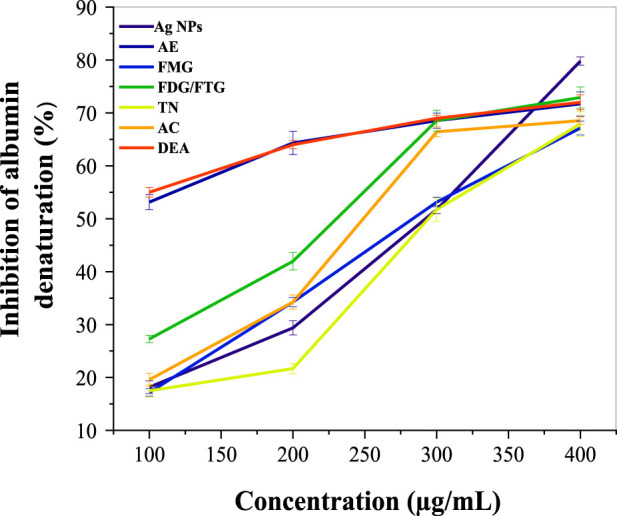
Comparative anti-inflammatory activity of Ag NPs, total aqueous extract (AE) and fractions from *H. lippii* (FMG, FDG/FTG, TN, and AC) at various concentrations (100–400 μg/mL), with Diclofenac (DEA) as the standard.

Figure 4 illustrates the anti-inflammatory activity of phytochemically modified Ag NPs and *H. lippii* fractions compared to diclofenac (DEA) as a standard (see Supplementary Table S2). At lower concentrations (100 μg/mL), the AE extract exhibited the highest inhibition (53.14%), surpassing Ag NPs (18.1%) and all fractions, including FMG (17.17%) and FDG/FTG (27.27%), closely matching diclofenac (55.1%). At 250 μg/mL, the AE extract continued to show the highest activity (64.35%), while Ag NPs (29.37%) and FDG/FTG (41.95%) exhibited moderate improvements. At 300 μg/mL, AE (68.53%) and FDG/FTG (68.53%) maintained their high activity, with Ag NPs showing significant enhancement (51.74%). However, at 400 μg/mL, Ag NPs reached their peak inhibition (79.8%), surpassing AE (71.75%) and all fractions, including FDG/FTG (72.9%) and TN (67.9%), with diclofenac showing comparable inhibition (72.63%). These findings demonstrate that while AE extract was the most effective at lower concentrations, Ag NPs exhibited superior inhibition at the highest concentration, indicating their potential for enhanced dose-dependent anti-inflammatory activity.

Previous studies suggest that the phytochemicals present in *H. lippii*, such as flavonoids and phenolic acids, contribute significantly to its anti-inflammatory properties, supporting the plant’s potential in traditional and complementary medicine (Saleh et al., 2021). The enhanced anti-inflammatory potential of Ag NPs has also been highlighted in the literature; where their unique properties, including increased bioavailability and a high surface-to-volume ratio, enable them to FMG effectively modulate oxidative stress pathways involved in the inflammatory process (Mohamed et al., 2024). Furthermore, studies have shown that various solvent fractions of plant extracts, exhibit moderate anti-inflammatory effects, while other fractions demonstrate relatively lower activities, indicating variations in the concentration of active compounds (Azevedo et al., 2010; Ramadwa et al., 2017).

### 3.5 Anti-hemolytic activity

**FIGURE 5:**
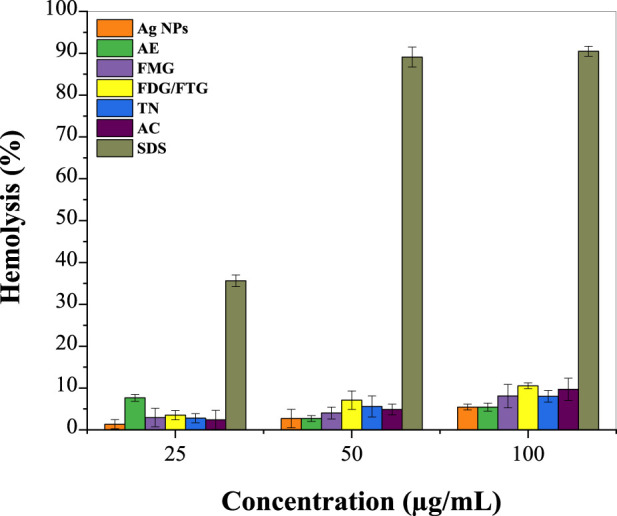
Percentage of hemolysis induced by Ag NPs, total aqueous extract (AE) and fractions from *H. lippii* (FMG, FDG/FTG, TN, and AC) at various concentrations, compared to SDS as the standard.

Figure 5 shows the hemolysis percentage induced by phytochemically modified Ag NPs and solvent fractions of *H. lippii* extract (FMG, FDG/FTG, TN, and AC) at various concentrations, compared to SDS as a standard (Supplementary Table S3). At 25 μg/mL, Ag NPs show minimal hemolytic activity (1.35%), while the solvent fractions exhibit slightly higher values, indicating low toxicity. At 50 μg/mL, hemolytic activity increases modestly for both Ag NPs and solvent fractions, with DF and NF showing higher percentages. At 100 μg/mL, hemolysis further increases, with Ag NPs reaching 5.43%, and solvent fractions, particularly FMG and NF, showing notable increases. In contrast, SDS demonstrates significantly higher hemolytic activity, highlighting the relatively lower cytotoxicity of the *H. lippii* fractions and suggesting a potentially safer profile for biomedical applications. Our results align with the findings of Zohra and Fawzia (2014), who studied the hemolytic activity of plant extracts and observed that *H. lippii* showed exceptionally low hemolysis rates, particularly at higher concentrations, with values of 1.91%, 3.8%, and 7.6% at 25, 50, and 100 μg/mL, respectively. Alsabri et al. (2012) demonstrated that *H. lippii* extract is a superior candidate with minimal hemolysis across all concentrations, emphasizing its potential as a natural and non-toxic alternative for therapeutic applications. Both *H. lippii* extract and Ag NPs combine therapeutic benefits with minimal adverse effects on red blood cells, supporting their promise for future biomedical applications in anti-inflammatory therapies, wound healing, and drug delivery systems (Chahardoli et al., 2023; Markandeywar and Narang, 2024).

### 3.6 Photoprotective activity

**FIGURE 6:**
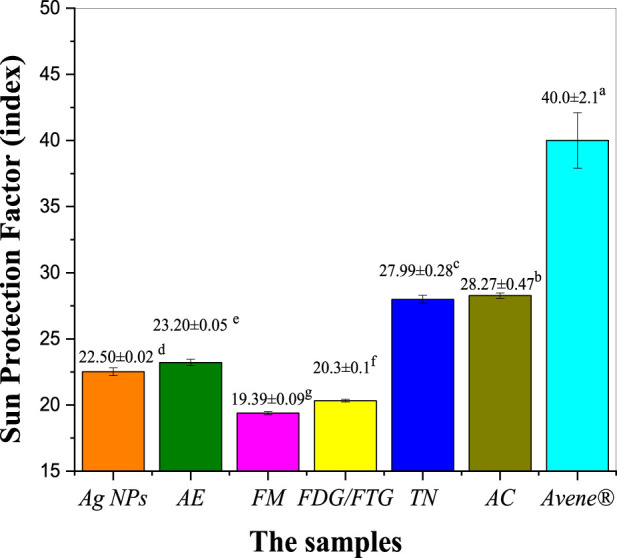
Assessing photoprotective properties of Ag NPs, total aqueous extract (AE) and fractions from *H. lippii* (FMG, FDG/FTG, TN, and AC) at concentration of 1 mg/mL; ^a-g^ Means with distinct letters in each column differ substantially (*p* < 0.05).

Figure 6 shows the photoprotective activity of photochemically modified Ag NPs and solvent fractions of *H. lippii* extract, assessed based on their SPF values and compared to the positive control, Avene^®^ (Supplementary Table S4 in the Supplementary Material). The crude extract of *H. lippii* demonstrated an SPF of 23.20, which is slightly lower than Avene^®^ (SPF 40.00), suggesting that the crude extract offers substantial protection against UV radiation. Ag NPs also exhibited good photoprotective activity, with an SPF of 22.50, indicating their potential for UV protection, comparable to that of the crude extract. Among the solvent fractions (TN and AC) showed superior SPF values of 27.99 and 28.27, respectively, both nearing the SPF of the crude extract. The FMG and FDG/FTG fractions displayed slightly lower SPF values of 19.39 and 20.30, reflecting moderate UV protection capabilities. These findings demonstrate that both Ag NPs and *H. lippii* fractions possess significant photoprotective potential, particularly for cosmetic and dermatological applications, with TN and AC fractions standing out for their effectiveness. While the Avene^®^ standard has the highest SPF, these materials could offer valuable alternatives for UV protection in skin care products.

Several studies have demonstrated the photoprotective capabilities of extract solvent fractions and Ag NPs. de Oliveira Raphaelli et al. (2021), reported that solvent fractions rich in antioxidants provide enhanced protection against UV radiation. Similarly, Rudrappa et al. (2023) (Rudrappa et al., 2023) emphasized the remarkable photoprotective properties of Ag NPs, attributing this to their capacity to absorb and reflect UV light, as well as their antioxidant effects that alleviate oxidative stress resulting from UV exposure. Conversely, some fractions exhibited lower photoprotective capacities, which may be linked to reduced concentrations of bioactive compounds, as highlighted by Dal Prá et al. (2017) in their analysis of fractions with diminished phenolic content and antioxidant activity. Variations in photoprotective efficacy among the tested samples are likely associated with differences in phenolic content and antioxidant strength, both of which have been identified as statistically significant factors (Lekmine et al., 2021; Nunes et al., 2018).

### 3.7 Antibacterial activity

Table 2 presents the antibacterial activity of Ag NPs, total aqueous extract (AE), and fractions from *H. lippii* based on inhibition zones, while Table 3 highlights Minimum Inhibitory Concentration (MIC) values. Ag NPs exhibited the highest antibacterial potency, with inhibition zones of 18.66–24.1 mm (250–1,000 μg/mL), particularly effective against *B. subtilis* and *S. aureus*. They also recorded the lowest MIC values of 0.046 mg/mL against *E. coli* and *S. aureus*. Among the fractions, the FMG fraction demonstrated strong antibacterial activity, with inhibition zones reaching up to 24.1 mm against *B. subtilis* at 1,000 μg/mL and 18.26 mm against *E. coli*, also showing favorable MIC values of 0.23 mg/mL against *S. aureus*. The TN fraction also displayed strong antibacterial effects, with inhibition zones of 26.67 mm against *B. subtilis* at 1,000 μg/mL, and MIC values ranging from 0.23 mg/mL to 0.46 mg/mL. In contrast, the FDG/FTG fraction exhibited moderate activity, with inhibition zones of 16–17 mm at 1,000 μg/mL and MIC values of 0.15 mg/mL against *Pseudomonas aeruginosa* and 0.46 mg/mL against *S. aureus* and *E. coli*. The AE fraction showed the weakest antibacterial effects, with inhibition zones of 16.3 mm at 1,000 μg/mL and the highest MIC values of 2.81 mg/mL for all bacterial strains. The AC fraction also demonstrated weaker activity, with inhibition zones of 16 mm at 1,000 μg/mL and relatively higher MIC values (0.93 mg/mL against *B. subtilis*).

**TABLE 2:** Antibacterial Activity of Ag NPs, total aqueous extract (AE) and fractions from *H. lippii* (FMG, FDG/FTG, TN, and AC) Against Gram-Positive and Gram-Negative Bacteria at Various Concentrations Using the Agar Well Diffusion Assay.

Sample	Concentration	Zone inhibition (mm)				
		*BS*	*EC*		*PA*	*SA*
Ag NPs	1000 μg/mL	18.66 ± 0.37^***^	12.00 ± 00^**^		18.33 ± 0.06^***^	14.00 ± 00^**^
	750 μg/mL	13.75 ± 0.035^**^	11.00 ± 00^**^		15.33 ± 0.06^***^	13.66 ± 0.05^**^
	500 μg/mL	12.5 ± 0.05^**^	10.66 ± 0.12^**^		14.00 ± 00^**^	13 ± 0.00^**^
	250 μg/mL	12 ± 0.1^**^	10.33 ± 0.12^**^		13.3 ± 0.16^**^	12.00 ± 0.1^**^
*AE*	1000 μg/mL	16.3 ± 0.3^***^	14.66 ± 0.06^**^		18.00 ± 00^***^	15.00 ± 00^***^
	750 μg/mL	14.33 ± 0.3^**^	12.33 ± 0.06^**^		17.00 ± 00^***^	14.667 ± 0.057^**^
	500 μg/mL	13.33 ± 0.05^**^	11.6 ± 0.05^**^		15.33 ± 0.06^***^	13.33 ± 0.15^**^
	250 μg/mL	10.06 ± 0.11^**^	11.00 ± 0.00^**^		14.33 ± 0.06	12.00 ± 0.1^**^
FMG	1000 μg/mL	24.1 ± 0.52^***^	18.26 ± 0.047^***^		16,00 ± 00^***^	17.00 ± 0.2^***^
	750 μg/mL	18.33 ± 0.29^***^	16.67 ± 0.05^***^		15,00 ± 00^***^	15.667 ± 0.057^***^
	500 μg/mL	18.09 ± 0.60^***^	15.67 ± 0.05^***^		14.00 ± 00^**^	13.667 ± 0.057^**^
	250 μg/mL	16.33 ± 0.32^***^	14.33 ± 0.12		14.00 ± 00^**^	11.00 ± 00^**^
FDG/FTG	1000 μg/mL	16 ± 0.1^***^	17.00 ± 0.1^***^		15.00 ± 00^***^	17.33 ± 0.06^***^
	750 μg/mL	15.6 ± 0.11^***^	13.00 ± 00^**^		14.33 ± 0.06^**^	17.00 ± 0.1^***^
	500 μg/mL	15.0 ± 0.1^***^	12.67 ± 0.05^**^		14.00 ± 0.1^**^	15.33 ± 0.16^***^
	250 μg/mL	12.6 ± 0.12^**^	8.633 ± 0.057^**^		13.33 ± 0.16^**^	14.00 ± 0.1^**^
TN	1000 μg/mL	26.67 ± 1.04^****^	17.00 ± 00^***^		22 ± 00^***^	24.00 ± 0.1^***^
	750 μg/mL	26.33 ± 0.05^****^	16.66 ± 0.057^***^		14.7 ± 0.8^**^	16.33 ± 0.16^***^
	500 μg/mL	25.66 ± 0.12^****^	16.00 ± 00^***^		14.00 ± 00^**^	15.00 ± 0.1^***^
	250 μg/mL	23.3 ± 0.2	14.00 ± 0.1^**^		11.00 ± 00^**^	14.66 ± 0.06
AC	1000 μg/mL	19.66 ± 0.40^***^	13.68 ± 0.05^**^		15.5 ± 0.05^***^	16.33 ± 0.06^***^
	750 μg/mL	17.33 ± 0.05^***^	13.00 ± 00^**^		14.667 ± 0.05^**^	14.00 ± 0.00^**^
	500 μg/mL	17.00 ± 0.01^***^	12.68 ± 0.05^**^		14.5 ± 0.05^**^	13.67 ± 0.05^**^
	250 μg/mL	14.33 ± 0.06^**^	11.33 ± 0.06^**^		11.67 ± 0.667^**^	12.00 ± 0.1^**^
DMSO	--	Nill	Nill		Nill	Nill
Amoxicillin	10 μg/mL	30 ± 0.00	25 ± 0.7		27.5 ± 0.7	30 ± 0.5
Cephalexin	30 μg/mL	32.5 ± 1.41	26 ± 0.3		32 ± 0.4	22 ± 0.7

*Bs: Bacillus subtilis* ATCC, 6633; *Ec: Escherichia coli ATCC*, 8737; *Pa: pseudomonas aeruginosa* ATCC, 9027; *Sa*: *Staphylococcus aureus* ATCC, 6538. DMSO: dimethyl sulfoxide. Inhibition zones > to 25 mm were considered as very strong (^****^), inhibition zones from 15 mm to 25 mm as strong (^***^), from 8 to 15 as moderated (^**^) and ≤8 as week activities (^*^) (Ismail et al., 2016).

**TABLE 3:** Minimum inhibitory concentration (MIC, mg/mL) of Ag NPs, total aqueous extract (AE) and fractions from *H. lippii* (FMG, FDG/FTG, TN, and AC) against various bacterial strains.

*Tested Samples*	*Tested Microorganism and MIC (mg/mL)*			
	*Bs*	*Ec*	*Pa*	*Sa*
Ag NPs	0.18 ± 0.01^a^	0.046 ± 0.04^a^	0.093 ± 0.02^a^	0.046 ± 0.01^a^
*AE*	2.81 ± 0.06^d^	2.81 ± 0.05^d^	2.81 ± 0.01^d^	2.81 ± 0.04^d^
FMG	0.46 ± 0.03^b^	0.46 ± 0.02^c^	0.46 ± 0.02^c^	0.23 ± 0.02^c^
FDG/FTG	0.46 ± 0.01^b^	0.46 ± 0.01^c^	0.15 ± 0.03^b^	0.15 ± 0.01^b^
TN	0.46 ± 0.04^b^	0.46 ± 0.05^c^	0.15 ± 0.04^b^	0.23 ± 0.01^c^
AC	0.93 ± 0.02^c^	0.23 ± 0.01^b^	0.46 ± 0.01^c^	0.23 ± 0.03^c^

a-d: means Waller Dunkan (*p* < 0.05). Fractions: FMG: flavonoid monoglycosides, FDG/FTG: flavonoid diglycosides and triglycosides; TN: tannins, AC: anthocyannins, Bs: *Bacillus subtilis* ATCC, 6633; Ec: *Escherichia coli* ATCC, 8737; Pa: *pseudomonas aeruginosa* ATCC, 9027; Sa: *Staphylococcus aureus* ATCC, 6538.

Overall, Ag NPs, along with the FMG and TN fractions, exhibited the strongest antibacterial properties, while AE and AC fractions showed significantly lower activity, indicating that both the concentration of the sample and the specific phytochemical content played a key role in enhancing the antibacterial effects. Previous studies have established a strong foundation for these observations, showing that bioactive compounds like tannins and flavonoids, found in the TN and FDG/FTG fractions, possess significant antimicrobial properties. Studies by Baydar et al. (2004), Medini et al. (2014) and Njateng et al. (2017) highlighted that these compounds inhibit bacterial growth by disrupting bacterial metabolism and cell membrane integrity (Pandey and Negi, 2018). Additionally, the effectiveness of Ag NPs in antibacterial applications has been well-documented, with Bhusal et al. (2024), Michailidu et al. (2022) and Khane et al. (2022) emphasizing their broad-spectrum activity, particularly against *Pseudomonas aeruginosa*, by generating reactive oxygen species (ROS) that damage bacterial membranes. Moreover, the synergistic effect between Ag NPs and phytochemicals has been noted in enhancing antimicrobial activity, particularly against resistant bacterial strains (AbuDalo et al., 2019).

## 4 Conclusion

This study investigates the photoprotective, anti-hemolytic, antibacterial, and anti-inflammatory properties of silver nanoparticles (Ag NPs) synthesized using *H. lippii* extract, and compares their efficacy with various extract fractions: total aqueous extract (AE), flavonoid monoglycosides (FMG), flavonoid diglycosides/triglycosides (FDG/FTG), tannins (TN), and anthocyanins (AC). The physicochemical analysis revealed that the Ag NPs are spherical, with an average size of 35 nm. HPLC analysis of AE identified key bioactive phytochemicals, such as caffeic acid, p-coumaric acid, and gallic acid, which contribute to the extract’s bioactivity. In antibacterial assays, Ag NPs exhibited superior activity compared to the extract fractions, with MICs of 0.2 mg/mL against *Pseudomonas aeruginosa* and 0.4 mg/mL against *B. subtilis*. These values were significantly lower than those observed for AE (MIC of 2.81 mg/mL) and fractions like TN (0.5 mg/mL against *S. aureus* and *E. coli*) and FDG/FTG (0.8 mg/mL against *Pseudomonas aeruginosa* and *B. subtilis*). Ag NPs thus displayed enhanced antibacterial potential compared to both the individual extract fractions and the controls. For anti-inflammatory effects, Ag NPs achieved 79.8% inhibition of albumin denaturation at 400 μg/mL, outperforming the AE (71.75%) and all extract fractions, such as TN (67.9%), FDG/FTG (72.9%), and even the standard drug, diclofenac (72.63%). Hemolytic activity for Ag NPs was minimal, with only 5.43% hemolysis at 100 μg/mL, much lower than the control sodium dodecyl sulfate (SDS), which caused 90.48% hemolysis. This indicates that Ag NPs are safer than the control for potential therapeutic use. In photoprotective assays, the TN fraction exhibited the highest SPF of 27.99, closely followed by the commercial sunscreen standard Avene^®^ (SPF 40). Ag NPs demonstrated an SPF of 22.50, which was lower than TN but still provided meaningful photoprotective potential. Overall, Ag NPs showed superior antibacterial, anti-inflammatory, and minimal hemolytic activity compared to the controls and standards, demonstrating their promising biomedical potential. The combined use of *H. lippii* extracts and Ag NPs shows synergistic effects, offering an advanced strategy for developing therapeutic agents with antibacterial, anti-inflammatory, and photoprotective properties. Further research is needed to explore the underlying mechanisms and practical applications of these compounds to fully unlock their biomedical potential.

## Data Availability

The original contributions presented in the study are included in the article/Supplementary Material, further inquiries can be directed to the corresponding author.
