# Supplementary material for: Phytochemical profiling and fractionation of Helianthemum lippii extract versus silver nanoparticle-modified extract: assessment of photoprotective, anti-hemolytic, antibacterial, and anti-inflammatory properties
Source: Front Chem. 2024 Dec 10;12:1508707. doi: 10.3389/fchem.2024.1508707 (PMC11666390; doi:10.3389/fchem.2024.1508707)
Supplement: Supplementary file 1 [file DataSheet1.PDF]

Phytochemical Profiling and Fractionation of *Helianthemum lippii* Extract Versus Silver Nanoparticle-Modified Extract: Assessment of Photoprotective, Anti-Hemolytic, Antibacterial, and Anti-Inflammatory Properties

Ibtissam Laib[1, 2], Djahra Ali Boutlilis[1,3] , Huda Alsaeedi[4], David Croun[5], Mikhael Bechelany[5,6], Ahmed Barhoum[7*]

[1] Department of Cellular and Molecular Biology, El Oued University, Algeria,

[2] Laboratory of Biodiversity and Biotechnology Applications in Agriculture, University of El Oued, 39000, Algeria, laib-ibtissam@univ-eloued.dz

[3] Laboratory of Biology, Environment and Health, Faculty of Natural and Life Sciences, University of El Oued, Algeria, djahra@yahoo.fr

[4] Department of Chemistry, College of Science, King Saud University, Riyadh, Saudi Arabia, halsaeedi@ksu.edu.sa

[5] Institut Européen des Membranes, IEM, UMR-5635, Univ Montpellier, ENSCM, CNRS, Place Eugene Bataillon, 34095 Montpellier, France, david.cornu@umontpellier.fr, mikhael.bechelany@umontpellier.fr

[6] Functional Materials Group, Gulf University for Science and Technology (GUST), Mubarak Al-Abdullah 32093, Kuwait

[7] NanoStruc Research Group, Chemistry Department, Faculty of Science, Helwan University, Cairo, 11795, Egypt

* Corresponding Author: ahmed.barhoum@science.helwan.edu.eg

Supplementary information

Table S1. Retention time and concentration of the phenolic compounds identified in the *H. lippii* aqueous extract.

**Phenolic compound**	**Class**	**Retention time (min)**	**Equation**	**Concentration (µg/mg extract)**
Caffeic acid	Hydroxycinnamic acid	16.27	y=42239x	444.81
p-Coumaric acid	Hydroxycinnamic acid	23.81	y=27977	663.77
Gallic acid	Hydroxybenzoic acid	5.29	y=54681x	9495.11
Vanillic acid	Hydroxybenzoic acid	15.53	y=20674	ND
Chlorogenic acid	Hydroxycinnamic acid	13.39	y=21665x	7107.24
Naringin	Flavonones	34.78	y=19379x	738.11
Rutin	Flavonols	28.37	y=1649x	ND
Quercetin	Flavonols	45.04	y=2142281x	1118.64
Vanillin	Hydroxybenzoic acid	21.46	y=9286x	ND

Table S2. Comparative Anti-inflammatory Activity of Ag NPs, total aqueous extract (AE) and fractions from *H. lippii* (FMG, FDG/FTG, TN, and AC) at various concentrations (100-400 µg/mL) comparing to DEA (positive control).

Concentration	% Inhibition of albumin denaturation						
	Ag NPs	AE	FMG	FDG&FTG	TN	AC	DEA
100 µg/mL	18.1 ± 0.4	53.14 ± 1.22	17.17 ± 0.71	27.27 ± 0.71	17.48 ± 1.23	19.58 ± 1.20	55.1 ± 0.7
250 µg/mL	29.37 ± 0.23	64.35 ± 1.34	34.2 ± 0.9	41.95 ± 1.65	21.67 ± 0.96	34.26 ± 1.34	64.4 ± 0.9
300 µg/mL	51.74 ± 0.41	68.53 ± 0.78	53.1 ± 0.9	68.53 ± 1.97	51.7 ± 2.2	66.43 ± 0.96	69.72 ± 0.56
400 µg/mL	79.8 ± 2.2	71.75 ± 0.78	67.1 ± 1.3	72.9 ± 1.9	67.9 ± 2.3	68.53 ± 0.70	72.63 ± 1.41

Table S3. Percentage of hemolysis induced by the Ag NPs, total aqueous extract (AE) and fractions from *H. lippii* (FMG, FDG/FTG, TN, and AC) at various concentrations compared to SDS (positive control).

**Concentration**	Percentage of haemolysis						
	**Ag NPs**	AE	FMG	FDG & FTG	TN	AC	SDS
25 µg/mL	1.35 ± 0.08	1.91 ± 0.02	2.95 ± 0.20	3.54 ± 0.12	2.80 ± 0.10	2.42 ± 0.28	35.62 ± 1.43
50 µg/mL	2.71 ± 0.71	3.80 ± 0.20	4.04 ± 0.11	7.09 ± 0.20	5.60 ± 0.50	4.86 ± 0.78	89.07 ± 2.49
100 µg/mL	5.43 ± 0.95	7.60 ± 0.70	8.08 ± 0.81	10.50 ± 0.70	8.01 ± 0.41	9.69 ± 0.66	90.48 ± 1.21

Table S4. Assessing photoprotective potential of Ag NPs, total aqueous extract (AE) and fractions from *H. lippii* (FMG, FDG/FTG, TN, and AC) at various concentrations comparing to Avene® (positive control); a-f: means Waller Dunkan ($p < 0.05$).

**Sample**	**SPF**
Ag NPs	22.50 ± 0.20^{e}
AE	23.20 ± 0.30^{d}
FMG	19.39 ± 0.11^{g}
FDG & FTG	20.3 ± 0.1^{f}
TN	27.99 ± 0.28^{c}
AC	28.27 ± 0.47^{b}
Avene®	40 ± 2.1^{a}

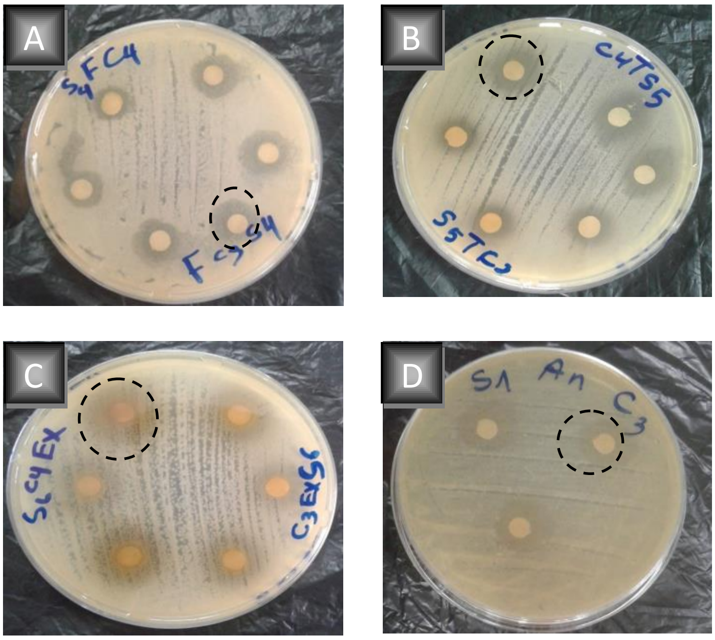

Figure S1: Zones of inhibition of *Helianthemum Lippii* L and their fractions against various bacterial strains **A:** Zones of inhibition for flavonoid monoglycosides fraction against *pseudomonas aeruginosa*, **B**: Zones of inhibition for fraction flavonoid diglycosides and triglycosides against *Escherichia coli*, **C**: Zones of inhibition for fraction anthocyannins against *Escherichia coli*, **D**: Zones of inhibition for aqueous extract of *H.lippii* against *Bacillus subtilis* , **E**: Zones of inhibition for fraction tannins against *Staphylococcus aureus*, **F**: Zones of inhibition for fraction Anthocyannins against *Bacillus subtilis*.

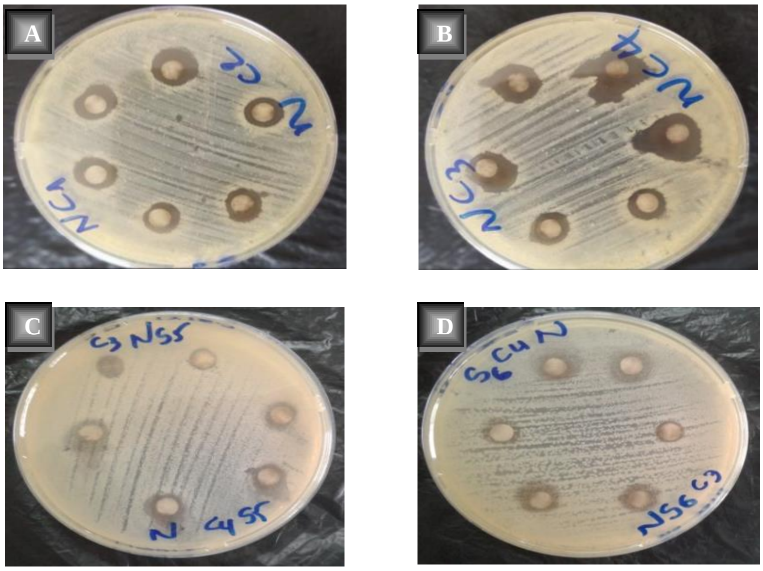

Figure S2: Antibacterial activity of synthesized Ag NPs at different strains bacteria indicated at numbers 1–4; 1: *Bacillus subtilis*, 2: *Escherichia coli*, 3: *Pseudomonas aeruginosa*, 4: *Staphylococcus aureus.*
